# Therapeutic efficacy and dermal safety of topical clove essential oil spray against bacterial, yeast, and fungal skin infections in rat models: A pilot non-inferiority study

**DOI:** 10.14202/vetworld.2026.4069-4084

**Published:** 2026-09-14

**Authors:** Eakachai Thongkham, Glenn Neville Borlace, Ranee Singh, Ketmanee Senaphan, Jareerat Aiemsaard

**Affiliations:** 1 Department of Pharmacology, Faculty of Veterinary Medicine, Mahanakorn University of Technology, Bangkok 10530, Thailand.; 2 Department of Pharmaceutical Chemistry, Faculty of Pharmaceutical Sciences, Khon Kaen University, Khon Kaen 40002, Thailand.; 3 Division of Pharmacology and Toxicology, Faculty of Veterinary Medicine, Khon Kaen University, Khon Kaen 40002, Thailand.; 4 Division of Physiology, Faculty of Veterinary Medicine, Khon Kaen University, Khon Kaen 40002, Thailand.

**Keywords:** clove essential oil, dermal safety, dermatophytosis, infectious dermatitis, Malassezia dermatitis, non-inferiority, pyoderma, topical antimicrobial

## Abstract

**Background and Aim:**

Infectious dermatitis caused by bacterial, yeast, and fungal pathogens is an important veterinary concern, while antimicrobial resistance and adverse effects associated with conventional therapies highlight the need for safe topical alternatives. Clove essential oil exhibits broad antimicrobial activity; however, controlled evidence of its therapeutic efficacy against multiple cutaneous pathogens remains limited. This pilot study evaluated the non-inferiority and dermal safety of a topical clove essential oil spray compared with standard treatments in rat models of pyoderma, *Malassezia *dermatitis, and dermatophytosis.

**Materials and Methods:**

Thirty Wistar Hannover rats were allocated among experimental skin infection models induced with *Staphylococcus pseudintermedius*, *Malassezia pachydermatis*, or *Microsporum canis*. For each model, four rats received 2.5% w/w clove essential oil spray, four received the standard treatment (2% w/v chlorhexidine for *S. pseudintermedius* or 2% w/v ketoconazole for *M. pachydermatis* and *M. canis*), and two served as negative controls. Treatments were applied topically twice daily for 15 days. Therapeutic efficacy was assessed on days 5, 10, and 15 using percentage reduction in clinical index score (CIS), pathogen reduction, and culture negativity. Non-inferiority was evaluated using a prespecified margin of −20% for CIS reduction. Dermal irritation was assessed using erythema and edema scores.

**Results:**

By day 10, clove essential oil spray was non-inferior to the standard treatments in all three infection models. CIS reductions for clove essential oil versus standard treatment were 59.03% versus 64.58% for *S. pseudintermedius*, 61.46% versus 61.81% for *M. pachydermatis*, and 55.56% versus 61.11% for *M. canis*. By day 15, CIS reductions reached 100%, 97.72%, and 80.56%, respectively, with corresponding standard treatment values of 100%, 100%, and 83.33%. Complete pathogen reduction was achieved for *S. pseudintermedius* and *M. pachydermatis*, whereas *M. canis* culture negativity reached 75% in both active-treatment groups. No erythema or edema was observed following repeated clove essential oil spray application.

**Conclusion:**

Topical 2.5% w/v clove essential oil spray demonstrated non-inferior therapeutic efficacy by day 10 and excellent dermal tolerability across bacterial, yeast, and fungal experimental skin infections. These proof-of-concept findings support further dose-optimization and randomized clinical studies in naturally infected companion animals.

## INTRODUCTION

Infectious dermatitis is a common clinical problem in companion animals and livestock. Although bacteria are the predominant etiological agents, yeasts and fungi also contribute substantially to cutaneous infections. In small-animal practice, *Staphylococcus pseudintermedius* is the bacterium most frequently associated with pyoderma [1]. The lipophilic yeast *Malassezia pachydermatis* is commonly associated with dermatitis and is also a major cause of otitis externa [2]. Dermatophytosis may be caused by several fungal species, including *Microsporum canis*, *Microsporum gypseum*, and *Trichophyton mentagrophytes*, and commonly manifests as alopecia, erythematous papules, scaling, and localized inflammation [3]. These infections are also a public health concern because antimicrobial-resistant pathogens are emerging and spreading. Methicillin-resistant *S. pseudintermedius* (MRSP) and methicillin-resistant *Staphylococcus aureus* (MRSA) are of particular concern because of their zoonotic potential, and a high prevalence of these resistant strains has been reported in veterinary hospitals in Thailand [4]. Furthermore, azole resistance in *M. pachydermatis* and dermatophytes has been associated with therapeutic failure and the persistence of chronic or subclinical infections. These infections may require higher drug doses, longer treatment, or alternative antimicrobial agents. Subclinically infected animals may also serve as reservoirs for transmission, thereby increasing the risk of pathogen dissemination, economic losses, and healthcare costs [5, 6].

Current therapeutic approaches for infectious dermatitis have several limitations. Systemically administered antimicrobials may cause adverse effects, including hypersensitivity reactions and potential hepatic or renal toxicity. Oral administration may also be challenging in veterinary practice because animals may resist medications with bitter or otherwise unpalatable characteristics. Topical antimicrobials and antiseptics provide an alternative by delivering active compounds directly to affected areas while potentially reducing systemic exposure; however, some topical formulations may cause cutaneous irritation. Moreover, accidental ingestion during grooming or licking may result in systemic exposure and associated toxicity, including renal toxicity associated with aminoglycosides and hepatotoxicity associated with ketoconazole [7]. These limitations, along with growing concerns about antimicrobial resistance, emphasize the need for effective topical alternatives with favorable safety profiles. Plant-derived products and herbal extracts represent potential sources of bioactive compounds that may be developed for this purpose.

Plant-derived extracts have demonstrated antimicrobial activity against a broad range of pathogens. Essential oils are particularly promising because their antimicrobial activity can involve multiple mechanisms, including disruption of microbial cell membranes and membrane-associated structures and interference with intracellular processes such as energy metabolism and protein function [8, 9]. Previous *in vitro* studies by our group showed that several essential oils from Thai medicinal plants inhibited common veterinary cutaneous pathogens, with clove essential oil showing strong activity against *S. pseudintermedius*, *M. pachydermatis*, and dermatophytes [10–12]. In addition to its antimicrobial properties, clove essential oil is relatively inexpensive and potentially suitable for large-scale production. Incorporation of botanical active compounds into topical spray formulations may offer additional practical advantages, including ease of application and uniform coverage of affected skin.

Despite encouraging *in vitro* findings, evidence supporting the therapeutic use of clove essential oil against infectious dermatitis *in vivo* remains limited. Previous studies have reported that topical clove essential oil reduced lesion size and alopecia associated with fungal infections in cattle and buffaloes [13, 14]. Similarly, a topical preparation containing eugenol, the principal bioactive constituent of clove essential oil, improved clinical outcomes in guinea pigs experimentally infected with *M. gypseum*, including reductions in inflammation, crust formation, and lesion diameter [15]. Clove essential oil and eugenol have also demonstrated anti-inflammatory activities in both *in vitro* and *in vivo* studies [16, 17]. Collectively, these findings indicate therapeutic potential; however, most evidence comes from *in vitro* investigations or studies focusing on individual pathogens or infection types.

A substantial translational gap therefore remains between the demonstrated *in vitro* antimicrobial activity of clove essential oil and its potential application as a practical topical treatment for veterinary infectious dermatitis. In particular, controlled evidence directly comparing a ready-to-use clove essential oil formulation with established topical therapies across bacterial, yeast, and fungal skin infections using standardized clinical and microbiological outcomes remains limited. Evidence regarding whether such a formulation can achieve therapeutic efficacy comparable to conventional treatments while maintaining acceptable dermal tolerability is also limited. Addressing these gaps is important before considering clove essential oil-based formulations for further clinical evaluation in companion animals. Rat models provide a standardized and reproducible experimental platform for preliminary proof-of-concept assessment before evaluation in naturally infected target animal populations.

Therefore, this pilot study aimed to evaluate the therapeutic efficacy and dermal safety of a topical clove essential oil spray using experimental rat models of pyoderma caused by *S. pseudintermedius*, *Malassezia* dermatitis caused by *M. pachydermatis*, and dermatophytosis caused by *M. canis*. The primary objective was to determine whether the clove essential oil spray was non-inferior to standard topical therapies, comprising chlorhexidine for bacterial infection and ketoconazole for yeast and fungal infections, based on reductions in clinical index scores. The secondary objectives were to evaluate pathogen reduction and microbial culture negativity during treatment and to assess dermal tolerability using standardized irritation scoring. This multi-pathogen non-inferiority approach was designed to provide proof-of-concept evidence for the potential development of clove essential oil spray as a topical therapeutic option for infectious dermatitis in veterinary medicine.

## MATERIALS AND METHODS

### Ethical approval

All experimental procedures involving animals were reviewed and approved by the Institutional Animal Care and Use Committee of Khon Kaen University, Thailand (Approval No. IACUC-KKU-81/63). All procedures were conducted in accordance with the ethical principles and guidelines for the care and use of animals in research established by the National Research Council of Thailand. Animal handling, housing, experimental infection, treatment, and clinical monitoring were performed with appropriate measures to minimize pain, distress, and unnecessary animal use. The experimental design incorporated the principles of replacement, reduction, and refinement (3Rs), including using fewer animals in the untreated control groups and integrating the dermal irritation assessment into the primary experimental cohort.

### Study period and location

The study was conducted from October 2021 to February 2024. The experimental procedures were conducted at the Northeast Laboratory Animal Center, Khon Kaen University, Khon Kaen, Thailand. The study included animal acclimatization, induction and confirmation of experimental dermatitis, topical treatment, clinical and microbiological assessments, and dermal irritation evaluation. 

### Study design

This pilot experimental study used a randomized, blinded, controlled non-inferiority design to evaluate the therapeutic efficacy and dermal safety of a 2.5% w/w clove essential oil spray in rat models of infectious dermatitis. Three pathogen-specific models were established using *S. pseudintermedius*, *M. pachydermatis*, and *M. canis*, representing pyoderma, *Malassezia* dermatitis, and dermatophytosis, respectively. Within each model, animals were allocated to the clove essential oil spray group, a standard treatment group, or a negative control group. Chlorhexidine spray (2% w/v; Chanjao Longevity Co., Ltd., Bangkok, Thailand) was used as the standard treatment for the bacterial model, whereas ketoconazole spray (2% w/v; Charoen Bhaesaj Lab Co., Ltd.) was used for the yeast and fungal models. Therapeutic efficacy was evaluated using changes in the clinical index score (CIS), pathogen load or culture negativity, and clinical resolution during the 15-day treatment period. Non-inferiority was assessed using a prespecified margin of −20% for the percentage reduction in CIS (%CIS reduction). Dermal tolerability was evaluated concurrently using standardized erythema and edema scoring.

### Preparation of clove essential oil spray

A topical liquid spray containing clove essential oil was prepared using an ethanol–Tween-80–isopropyl myristate vehicle, modified from the formulation described by Worawong *et al.* [18]. Clove essential oil (2.5% w/w; Thai-China Flavors and Fragrances Industry Co., Ltd., Ayutthaya, Thailand) was blended with absolute ethyl alcohol (5–15% w/w; Merck, Darmstadt, Germany) and Tween-80 (5–15% w/w; Chemipan Corporation Co., Ltd., Bangkok, Thailand). The mixture was homogenized using a vortex mixer for 5 min to ensure uniform incorporation of the lipophilic components. Subsequently, isopropyl myristate (67.5–87.5% w/w; Chemipan Corporation Co., Ltd., Bangkok, Thailand) was added as the carrier, and the mixture was homogenized.

Because of commercial confidentiality, the exact concentrations of the excipients are reported as functional concentration ranges. Gas chromatography–mass spectrometry (GC-MS) characterization of the same batch of essential oil had previously demonstrated a composition of 98.87% eugenol and 1.13% trans-caryophyllene [12]. Because the formulation was non-aqueous and contained no water phase, pH determination was not applicable. A single batch of the prototype spray, sufficient for all experiments, was freshly prepared under standardized conditions to minimize interbatch variability and was stored in light-protected amber glass containers at 4°C throughout the study.

### Experimental animals and sample size calculation

Eight-week-old male specific pathogen-free (SPF) Wistar Hannover GALAS rats [BrlHan:WIST@Jcl(GALAS)], weighing 200–300 g, were used as the experimental model. The animals were obtained from Nomura Siam International Co., Ltd., Thailand, and all experimental procedures were conducted at the Northeast Laboratory Animal Center, Khon Kaen University. Before experimentation, the rats were acclimatized for 7 days. Animals were individually housed in ventilated cages (IVC; 26.6 × 42.5 × 21 cm) containing wood-shaving bedding, which was replaced every 2–3 days. Barrier-maintained SPF conditions were maintained at 23 ± 2°C, 50%–60% relative humidity, 350–400 lux light intensity, and a 12-h light/12-h dark cycle, with controlled ventilation, low ambient noise, and backup power and water systems. Commercial pellet feed and clean water were provided *ad libitum*. Animals exhibiting systemic illness or severe stress unrelated to the experimental infection were predefined for exclusion; however, no animals met these exclusion criteria.

The sample size was calculated for a non-inferiority test of continuous outcomes using N4STUDIES software (version 1.4.1; Chetta Ngamjarus and Porjai Pattanittum, Khon Kaen, Thailand).

n =\(\frac{{\left({Z}_{1-\alpha }+{Z}_{1-\beta }\right)}^{2}{\sigma }^{2}(1+\frac{1}{κ})}{{(\varepsilon -\delta )}^{2}}\)

Because clinical data on clove essential oil treatment for veterinary dermatological conditions were limited, the power analysis assumptions were based on previous efficacy studies of botanical preparations [15, 19]. The calculation assumed a standard deviation (σ) of 5% for %CIS reduction, a one-sided significance level (α) of 0.05 (Z₁−α = 1.645), statistical power of 80% (β = 0.20; Z₁−β = 0.84), an expected mean difference (ε) of 10% between the experimental and standard treatment groups, a non-inferiority margin (δ) of 20%, and a 1:1 allocation ratio (κ = 1).

Based on these assumptions, four rats were required for each experimental and standard treatment group within each pathogen model. To minimize the number of animals exposed to untreated experimental infections in accordance with animal-welfare considerations, the negative control group contained two rats per pathogen model and was used primarily to provide qualitative and quantitative baseline validation of active infection. Thus, each pathogen model included 10 rats, for a total experimental cohort of 30 rats. The distribution of animals among the experimental groups is presented in Table 1.

**Table 1:** Distribution of experimental animals among infectious dermatitis models and treatment groups

**Infectious dermatitis model**	**Treatment/test group**	**Number of rats (n)**
*Staphylococcus * *pseudintermedius*	Clove essential oil spray	4
	Chlorhexidine spray (positive control)	4
	Negative control (untreated)	2
*Malassezia * *pachydermatis*	Clove essential oil spray	4
	Ketoconazole spray (positive control)	4
	Negative control (untreated)	2
*Microsporum * *canis*	Clove essential oil spray	4
	Ketoconazole spray (positive control)	4
	Negative control (untreated)	2
Total		30

The dermal irritation assessment was conducted concurrently using a subset of eight rats from the primary cohort of 30 rats; four received clove essential oil spray and four received normal saline on non-infected control skin sites.

### Microbial inoculum preparation

The microorganisms used to establish the experimental dermatitis models were *S. pseudintermedius* American Type Culture Collection (ATCC) 49051, *M. pachydermatis* ATCC 14522, and *M. canis* Department of Medical Sciences Thailand (DMST) 29297. *S. pseudintermedius* was inoculated into Mueller–Hinton broth (MHB; BD Difco™, Becton, Dickinson and Company, Le Pont-de-Claix, France) and incubated at 37°C for 24 h. *M. pachydermatis* was inoculated into Sabouraud dextrose broth (SDB; BD Difco™, Becton, Dickinson and Company) and incubated at 37°C for 48 h. After incubation, the microbial suspensions were standardized using optical density (OD) measurements and adjusted to final concentrations of 10⁶–10⁷ colony-forming units (CFU)/mL.

For the dermatophyte model, *M. canis* was cultured on potato dextrose agar (PDA; BD Difco™, Becton, Dickinson and Company) at 30°C for 14–21 days to obtain adequate sporulation. Fungal elements were harvested by flooding the agar surface with normal saline and gently scraping the culture using a sterile L-shaped glass spreader. The resulting suspension was transferred to a sterile tube and adjusted to 10⁴–10⁵ CFU/mL. The final viable counts of all standardized inocula were confirmed using the standard plate-count method [20–22].

### Induction of experimental dermatitis

Before experimental procedures, all rats were weighed and anesthetized with 2%–2.5% isoflurane in oxygen. To prevent hypothermia during induction, the animals were maintained on a heating pad at 37°C [23]. Two 4-cm² areas, located on the dorsal-thoracic and abdominal regions, with one site on each side, were shaved using a sterile single-use razor. The skin barrier was disrupted using a tape-stripping technique consisting of 10 consecutive applications and removals of adhesive tape to compromise the stratum corneum. The prepared areas were subsequently cleansed with sterile normal saline and dried with sterile cotton gauze.

A 2.25-cm² rectangular area (1.5 × 1.5 cm) was demarcated at the center of each shaved site using white petrolatum. Animals were randomly assigned to inoculation with *S. pseudintermedius*, *M. pachydermatis*, or *M. canis*. One prepared site received 50 µL of the corresponding microbial suspension, whereas the contralateral site served as an uninfected control and received an equivalent volume of sterile normal saline. After the inoculum dried, the sites were covered with sterile gauze and surgical tape [24–26]. Clinical signs were monitored daily during the development of experimental dermatitis.

The severity of experimentally induced lesions was evaluated using the CIS. Because the three pathogens produced different clinical manifestations, pathogen-specific parameters were used. For each animal, the CIS was the sum of three clinical parameters, each scored on a 4-point scale: 0 = absent, 1 = mild, 2 = moderate, and 3 = severe [27]. For the *S. pseudintermedius* pyoderma model, erythema, purulent exudate, and crust formation were assessed. For the *M. pachydermatis* *Malassezia* dermatitis model, erythema, greasy/hyperkeratotic appearance, and scaling were assessed. For the *M. canis* dermatophytosis model, erythema, scaling, and skin ulceration were assessed.

The maximum CIS was 9, representing the greatest combined severity of the three evaluated clinical parameters. All clinical assessments were performed by a single trained investigator who was blinded to treatment allocation to minimize interobserver variability. Before the experiment, intraobserver consistency was established through standardized calibration using representative lesion images. Lesions were photographed systematically on designated assessment days under standardized lighting, distance, and angle, with a calibrated scale bar included for visual verification.

### Confirmation and quantification of pathogens

Experimental infection was considered established when the CIS reached ≥4, which occurred 7–14 days after microbial inoculation. Pathogen recovery and quantification were performed according to the microorganism used in each experimental model.

For *S. pseudintermedius*, lesion samples were collected using sterile cotton swabs, suspended in normal saline, and transported at 4°C. Samples were cultured on HIMEDIA® Staphylococcus Agar No. 110 w/Azide (HiMedia Laboratories Pvt. Ltd., Thane, India), a selective medium for coagulase-positive staphylococci, and incubated at 37°C for 24 h. Bacterial density was expressed as CFU/cm². The limit of detection was 10 CFU/cm², and values below this threshold were considered non-detectable [21].

For *M. pachydermatis*, swab samples were inoculated onto Mycosel™ Agar (Becton, Dickinson and Company) and incubated at 37°C for 24 h. Identity was confirmed by growth on HIMEDIA® Dixon Agar (HiMedia Laboratories Pvt. Ltd.) at 32°C, 37°C, and 40°C and by a positive Tween utilization test using Tweens 20, 40, 60, and 80 (Chanjao Longevity Co., Ltd.). Microscopic examination following lactophenol cotton blue staining at 400–1,000× magnification was used to identify the characteristic oval or peanut-shaped budding cells (1–2 × 2–4 µm). Yeast density was expressed as CFU/cm², with a limit of detection of 10 CFU/cm² [28].

For *M. canis*, skin scrapings and hair shafts collected from lesion margins were inoculated onto dermatophyte test medium (HiMedia Laboratories Pvt. Ltd.) and incubated at 25°C–30°C for 5–21 days. Cultures were monitored for characteristic white, cottony-to-powdery colonies and a change in medium color from yellow to red. Identification was confirmed by lactophenol cotton blue staining, demonstrating characteristic spindle-shaped, thick-walled macroconidia containing 6–15 internal cells and frequently exhibiting a curved apex [28]. Because dermatophyte growth varies by phase, *M. canis* was assessed qualitatively as culture-positive or negative rather than by quantitative enumeration.

### Treatment protocol and clinical evaluation

Within each infection model, rats were randomly allocated to treatment groups using a simple lottery method in which folded, numbered slips corresponding to cage numbers were drawn. To reduce allocation and assessment bias, an independent assistant not involved in outcome evaluation and assigned anonymized codes (Treatments A, B, and C) to the treatment preparations. Investigators responsible for CIS assessment and microbial culture quantification remained blinded to treatment identity throughout the study.

All treatments were administered topically at approximately 0.1 g/cm² of lesion area twice daily, at 8–12 h intervals, for 15 days. In the standard treatment group, animals with *S. pseudintermedius* infection received 2% w/v chlorhexidine spray, whereas those with *M. pachydermatis* or *M. canis* infection received 2% w/v ketoconazole spray. Animals in the experimental group received the developed 2.5% w/w clove essential oil spray. Animals in the negative control group received normal saline to provide a baseline for untreated disease progression.

Therapeutic outcomes were assessed at 5-day intervals on days 5, 10, and 15. Outcomes included CIS, pathogen load, microbial culture status, and clinical resolution. Pathogen load was quantitatively evaluated for *S. pseudintermedius* and *M. pachydermatis*, whereas *M. canis* was assessed according to culture positivity or negativity. For each animal, percentage reductions in CIS and pathogen load were calculated relative to pretreatment values, and group means were subsequently determined. A reduction in CIS of ≥50% from baseline was considered a clinically meaningful response [29].

### Dermal irritation assessment

In accordance with the reduction and refinement components of the 3Rs principles, the dermal irritation assessment was integrated into the existing experimental cohort rather than using an additional group of animals. The assessment was performed on non-infected control skin sites after the skin barrier recovered from the initial tape-stripping procedure and was conducted concurrently with the therapeutic experiments. Eight rats were randomly selected, of which four received the clove essential oil spray and four received normal saline.

The formulations were applied topically at 0.1 g/cm² twice daily at 8–12 h intervals for four consecutive days. Dermal reactions were evaluated 24 h after the final application using a protocol adapted from the Organization for Economic Co-operation and Development (OECD) Guideline 404 [30] and a previous study [19]. Erythema and edema were each scored on a 5-point scale ranging from 0 to 4.

Erythema was scored as follows: 0 = no erythema; 1 = very slight erythema (barely perceptible); 2 = well-defined erythema; 3 = moderate erythema; and 4 = severe erythema (beet redness) to slight eschar formation (injuries in depth). Edema was scored as follows: 0 = no edema; 1 = very slight edema (barely perceptible); 2 = slight edema (edges of the area well-defined by definite raising); 3 = moderate edema (raised approximately 1 mm); and 4 = severe edema (raised >1 mm and extending beyond the area of exposure). The cumulative scores were used to determine the primary irritation index and evaluate the dermal tolerability of the clove essential oil formulation.

### Statistical analysis

All statistical analyses were performed using IBM SPSS Statistics version 28 (IBM Corp., Armonk, NY, USA). Statistical significance was set at p < 0.05. Data distribution was assessed for normality using the Shapiro–Wilk test. A reduction in CIS of ≥50% relative to baseline was considered a clinically meaningful response [29].

Within each infection model, longitudinal changes in %CIS reduction were analyzed using the Friedman test for repeated-measures, followed by Dunn–Bonferroni post hoc pairwise comparisons to account for multiple testing. For normally distributed pathogen-density data, repeated-measures analysis of variance was used, followed by Bonferroni-adjusted post hoc comparisons.

Non-inferiority of the clove essential oil spray relative to the corresponding standard treatment was assessed using a prespecified margin (δ) of −20% for %CIS reduction, based on criteria reported in a comparable dermatological non-inferiority study [27]. Non-inferiority was concluded when the lower bound of the 95% confidence interval (CI) for the difference in %CIS reduction between the experimental and standard treatment groups (experimental minus standard treatment) exceeded the prespecified non-inferiority margin.

Statistical analyses were conducted using a complete-case, per-protocol (PP) approach. All animals completed the 15-day study without mortality, dropout, or missing observations; therefore, data imputation was not required. For the dermal irritation assessment, paired ordinal scores obtained from treated and control sites were compared at corresponding assessment points using the Wilcoxon signed-rank test.

## RESULTS

### Induction of experimental dermatitis and clinical progression

Initial clinical manifestations following microbial inoculation were mild across all three infection models. During the first 7 days after inoculation, lesion development was insufficient for therapeutic evaluation, with CIS values ranging from 1 to 3 out of a maximum score of 9 (Figure 1). By day 14, however, lesions had progressed in all inoculated animals and reached the predefined threshold for therapeutic evaluation (Figure 2). At this time point, all inoculated rats were culture-positive for their respective pathogens.

**Figure 1:**
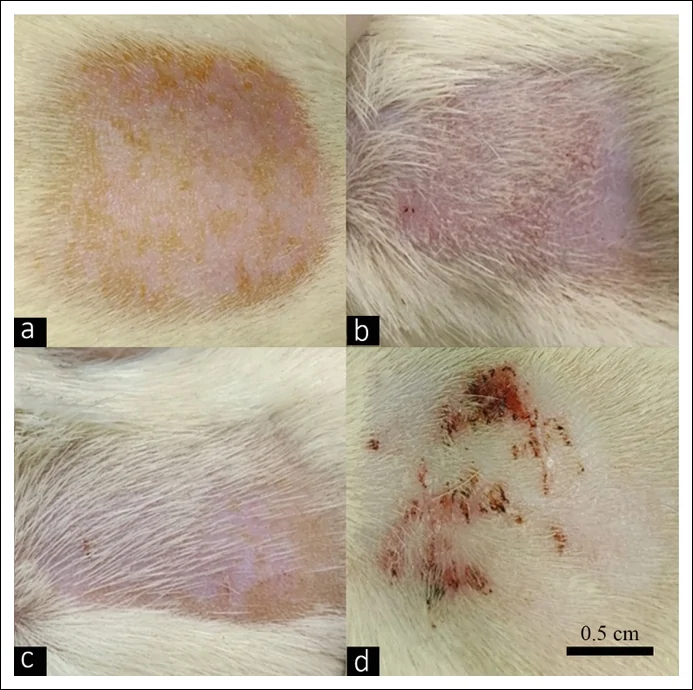
Clinical manifestations of experimental dermatitis models at baseline and 7 days after microbial inoculation. (a) Control site before microbial inoculation; (b) lesion induced by *Staphylococcus pseudintermedius* (CIS = 2/9); (c) lesion induced by *Malassezia pachydermatis* (CIS = 1/9); and (d) lesion induced by *Microsporum canis* (CIS = 3/9). CIS = Clinical index score.

**Figure 2:**
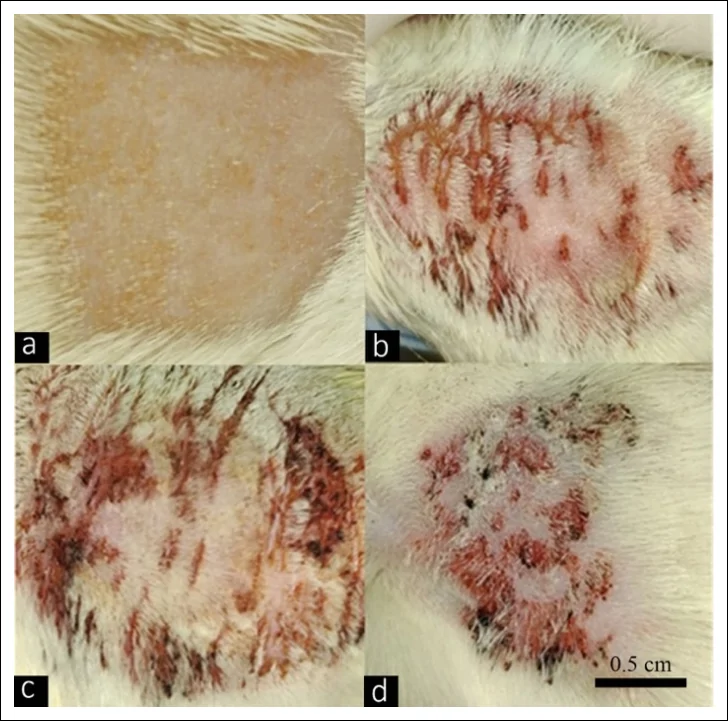
Progression of experimental infectious dermatitis lesions at 14 days after microbial inoculation. (a) Uninfected control site showing no observable lesions; (b) severe pyoderma induced by *Staphylococcus pseudintermedius* (CIS = 9/9); (c) *Malassezia* dermatitis induced by *Malassezia pachydermatis* (CIS = 9/9); and (d) dermatophytosis induced by *Microsporum canis* (CIS = 9/9). CIS = Clinical index score.

Before treatment initiation, the mean CIS values were 8.40 ± 0.52 for the *S. pseudintermedius* model, 8.50 ± 0.53 for the *M. pachydermatis* model, and 9.00 ± 0.00 for the *M. canis* model. Within each infection model, no statistically significant differences were observed among treatment groups in baseline CIS or initial pathogen load, indicating comparable pretreatment conditions across the groups (Table 2). In contrast, the contralateral uninfected control sites remained clinically normal throughout the 14-day induction period, with no observable lesions and negative microbial culture results.

**Table 2:** Baseline clinical index scores, microbial culture results, and pathogen loads of experimental lesions at day 14 after inoculation.

**Inoculated pathogen**	**Treatment group**	**CIS**	**Culture positivity (%)**	**Pathogen load (CFU/cm²)**
*Staphylococcus * *pseudintermedius*	Clove essential oil spray	8.50 ± 0.58	100.00 ± 0.00	1.21 ± 1.90 × 10⁵
	Standard spray	8.50 ± 0.58	100.00 ± 0.00	1.70 ± 1.67 × 10⁵
	Negative control	8.00 ± 0.00	100.00 ± 0.00	1.33 ± 1.82 × 10⁵
	Overall mean ± SD	8.40 ± 0.52	100.00 ± 0.00	1.43 ± 1.60 × 10⁵
	p-value*	0.472	NA	0.441
*Malassezia * *pachydermatis*	Clove essential oil spray	8.75 ± 0.50	100.00 ± 0.00	2.74 ± 1.43 × 10²
	Standard spray	8.25 ± 0.50	100.00 ± 0.00	2.22 ± 1.50 × 10²
	Negative control	8.50 ± 0.71	100.00 ± 0.00	2.19 ± 1.83 × 10²
	Overall mean ± SD	8.50 ± 0.53	100.00 ± 0.00	2.42 ± 1.37 × 10²
	p-value*	0.407	NA	0.683
*Microsporum * *canis*	Clove essential oil spray	9.00 ± 0.00	100.00 ± 0.00	NA
	Standard spray	9.00 ± 0.00	100.00 ± 0.00	NA
	Negative control	9.00 ± 0.00	100.00 ± 0.00	NA
	Overall mean ± SD	9.00 ± 0.00	100.00 ± 0.00	NA
	p-value*	NA	NA	NA

Data are presented as mean ± SD. CIS = Clinical index score; CFU = Colony-forming unit; NA = Not applicable. Numerical pathogen quantification was not performed for *M. canis*. Statistical tests could not be performed for *M. canis* or culture positivity because all observations had zero variance. Standard sprays comprised chlorhexidine for *S. pseudintermedius* and ketoconazole for *M. pachydermatis* and *M. canis*. The negative control received normal saline. *Differences among the three groups were analyzed using the Kruskal–Wallis test.

### Therapeutic efficacy in experimental models

Across the three infection models, the evaluated therapeutic outcomes, including %CIS reduction, %pathogen reduction, and %culture negativity, differed significantly between the active-treatment groups and the negative control group (p < 0.05; Table 3 and Figures 3–5). Both clove essential oil spray and the corresponding standard treatments reduced clinical severity and pathogen burden or increased culture negativity during treatment. In contrast, animals receiving normal saline showed little or no improvement in CIS and an increase in pathogen-density where quantitative enumeration was performed.

**Table 3:** Percentage reductions in clinical index score and pathogen load across experimental infectious dermatitis models.

**Follow-up**	**Treatment group**	***Staphylococcus *** ***pseudintermedius*** ** pyoderma: %CIS reduction**	***S. *** ***pseudintermedius*** ** pyoderma: %pathogen reduction**	***Malassezia *** ***pachydermatis*** ** dermatitis: %CIS reduction**	***M. *** ***pachydermatis*** ** dermatitis: %pathogen reduction**	***Microsporum *** ***canis*** ** dermatophytosis: %CIS reduction**	***M. *** ***canis*** ** dermatophytosis: %culture ** **negativity**
1 (day 5)	Clove essential oil spray	17.71 ± 6.93ᵃ	42.81 ± 45.70ᵃ	26.04 ± 9.85ᵃ	99.35 ± 0.51ᵃ	27.78 ± 6.42ᵃ	0.00*
	Standard spray	23.61 ± 9.21ᵃ	53.93 ± 80.47ᵃ	37.85 ± 9.17ᵇ	99.58 ± 0.53ᵃ	33.33 ± 9.07ᵃ	0.00
	Negative control	6.25 ± 8.84ᵇ	−382.93 ± 529.24ᵇ	0.00 ± 0.00ᶜ	−28.85 ± 100.63ᵇ	5.56 ± 7.86ᵇ	0.00
2 (day 10)	Clove essential oil spray	59.03 ± 4.01ᵃ	100.00 ± 0.01ᵃ	61.46 ± 7.89ᵃ	100.00 ± 0.00ᵃ	55.56 ± 9.07ᵃ	25.00*
	Standard spray	64.58 ± 2.41ᵃ	100.00 ± 0.00ᵃ	61.81 ± 4.61ᵃ	100.00 ± 0.00ᵃ	61.11 ± 6.42ᵃ	50.00
	Negative control	12.50 ± 0.00ᵇ	−185.72 ± 23.91ᵇ	11.11 ± 15.71ᵇ	−59.62 ± 127.82ᵇ	5.56 ± 7.86ᵇ	0.00
3 (day 15)	Clove essential oil spray	100.00 ± 0.00*	100.00 ± 0.00ᵃ	97.72 ± 5.56ᵃ	100.00 ± 0.00ᵃ	80.56 ± 5.56ᵃ	75.00*
	Standard spray	100.00 ± 0.00	100.00 ± 0.00ᵃ	100.00 ± 0.00ᵃ	100.00 ± 0.00ᵃ	83.33 ± 6.42ᵃ	75.00
	Negative control	0.00 ± 0.00	−220.35 ± 314.69ᵇ	17.36 ± 6.87ᵇ	−34.23 ± 135.44ᵇ	16.67 ± 7.86ᵇ	0.00

Data are presented as mean ± SD. CIS = Clinical index score. Standard sprays comprised chlorhexidine for *S. pseudintermedius* and ketoconazole for *M. pachydermatis* and *M. canis*. The negative control received normal saline. ᵃ,ᵇ,ᶜValues with different superscript letters within the same column at each follow-up differ significantly (p < 0.05). *Statistical comparison could not be performed because the relevant groups had zero variance.

**Figure 3:**
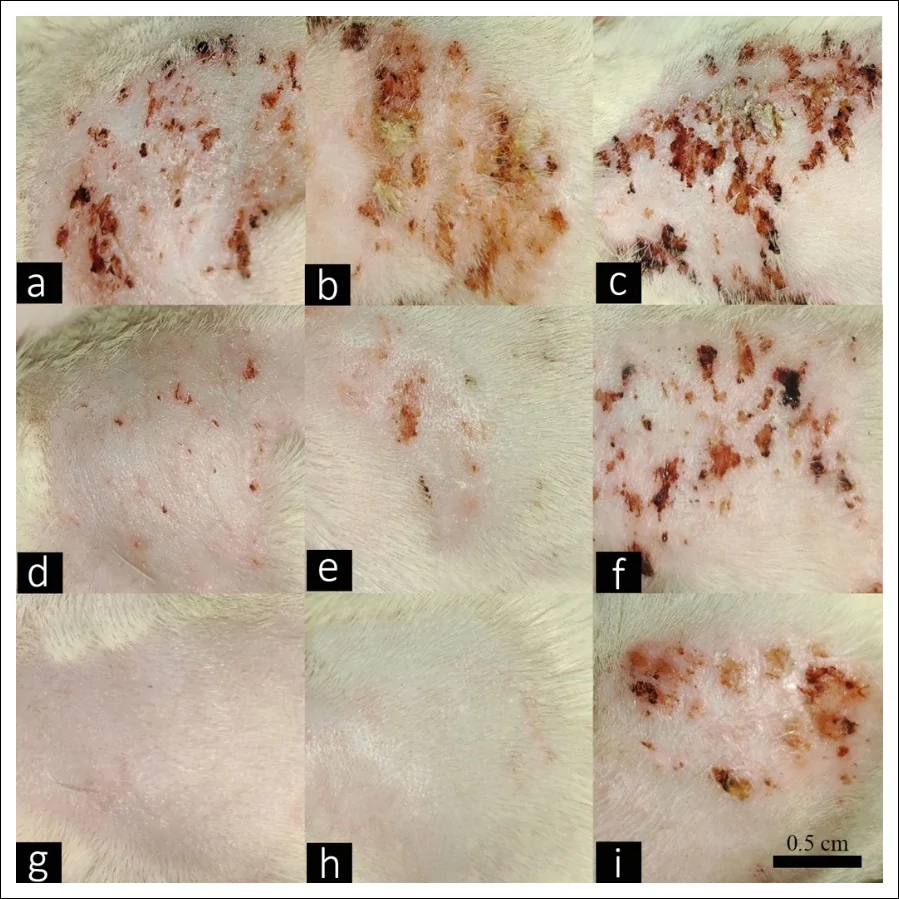
Progression of cutaneous lesions in the *Staphylococcus pseudintermedius* pyoderma model during treatment. (a, d, g) Chlorhexidine spray on days 5, 10, and 15, respectively; (b, e, h) clove essential oil spray on days 5, 10, and 15, respectively; and (c, f, i) negative control (normal saline) on days 5, 10, and 15, respectively.

**Figure 4:**
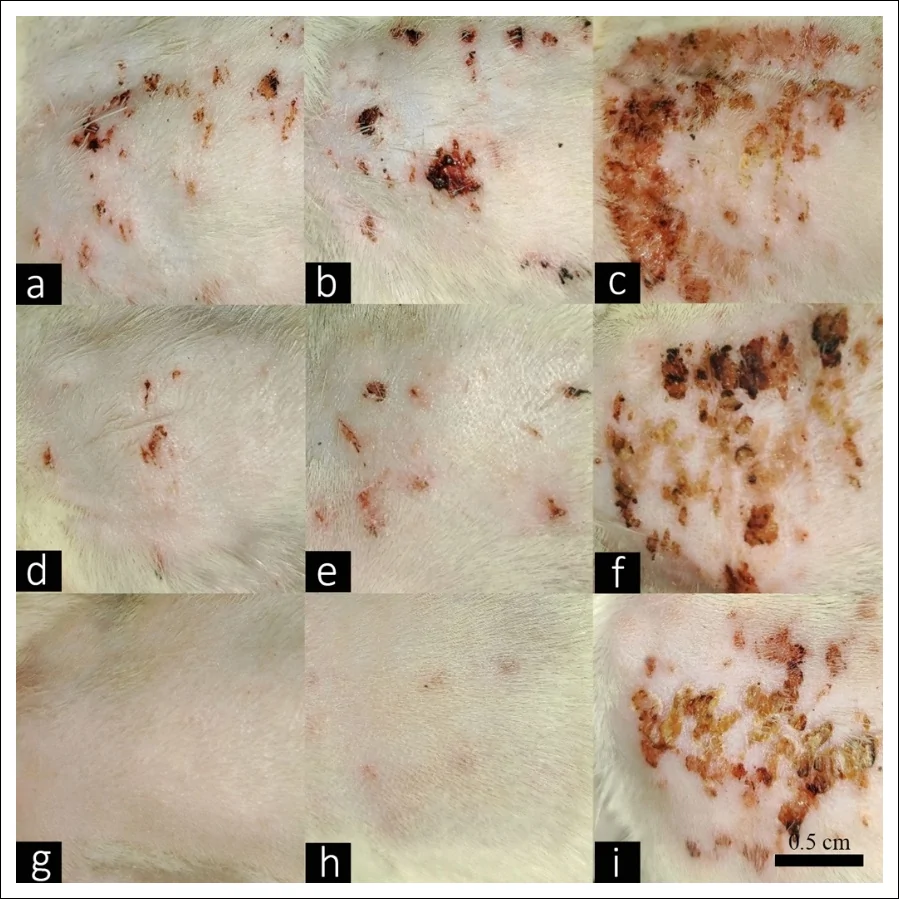
Progression of cutaneous lesions in the *Malassezia pachydermatis* dermatitis model during treatment. (a, d, g) Ketoconazole spray on days 5, 10, and 15, respectively; (b, e, h) clove essential oil spray on days 5, 10, and 15, respectively; and (c, f, i) negative control (normal saline) on days 5, 10, and 15, respectively.

In the *S. pseudintermedius* pyoderma model, both clove essential oil spray and chlorhexidine spray achieved the predefined clinical response criterion of ≥50% CIS reduction by the second follow-up on day 10. At this time point, the %CIS reduction was 59.03 ± 4.01% in the clove essential oil group and 64.58 ± 2.41% in the chlorhexidine group. Both active-treatment groups also achieved negative culture results by day 10. By the final follow-up on day 15, the CIS reduction reached 100.00 ± 0.00% in both groups, indicating complete clinical resolution.

**Figure 5:**
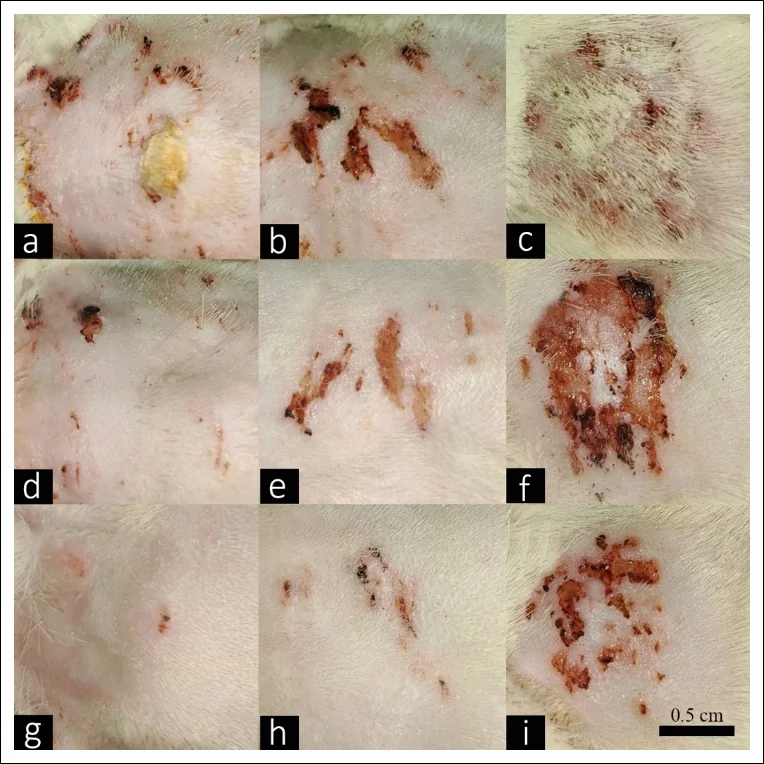
Progression of cutaneous lesions in the *Microsporum canis* dermatophytosis model during treatment. (a, d, g) Ketoconazole spray on days 5, 10, and 15, respectively; (b, e, h) clove essential oil spray on days 5, 10, and 15, respectively; and (c, f, i) negative control (normal saline) on days 5, 10, and 15, respectively.

In the *M. pachydermatis* *Malassezia* dermatitis model, both clove essential oil spray and ketoconazole spray achieved the predefined clinical response criterion and negative culture results from day 10 onward. At day 10, the %CIS reduction was 61.46 ± 7.89% in the clove essential oil group and 61.81 ± 4.61% in the ketoconazole group. By day 15, the %CIS reduction reached 97.72 ± 5.56% in the clove essential oil group and 100.00 ± 0.00% in the ketoconazole group.

In the *M. canis* dermatophytosis model, both active treatments achieved the predefined clinical response criterion by day 10. At this time point, the %CIS reduction was 55.56 ± 9.07% in the clove essential oil group and 61.11 ± 6.42% in the ketoconazole group. By day 15, these values had increased to 80.56 ± 5.56% and 83.33 ± 6.42%, respectively. Culture negativity at day 15 was 75% in both active-treatment groups.

### Non-inferiority analysis of clove essential oil spray

Non-inferiority of the clove essential oil spray relative to the corresponding standard treatments was evaluated using the prespecified margin of δ = −20% for %CIS reduction. In the *S. pseudintermedius* pyoderma model, the clove essential oil spray did not meet the non-inferiority criterion at the first follow-up on day 5 because the lower bound of the 95% CI for the between-group difference in %CIS reduction was below the prespecified margin. By day 10, however, the lower bound of the 95% CI exceeded the non-inferiority margin, indicating non-inferiority to chlorhexidine. At day 15, both treatments produced identical %CIS reduction values (Table 4).

**Table 4:** Non-inferiority analysis of clove essential oil spray versus chlorhexidine spray in the Staphylococcus pseudintermedius pyoderma model.

**Follow-up**	**Treatment**	**%CIS reduction (mean ± SD)**	**Non-inferiority margin**	**Mean difference**	**SE of difference**	**95% CI, lower**	**95% CI, upper**	**Result**
1 (day 5)	Clove essential oil spray	17.71 ± 6.93	−4.72	5.90	5.76	−8.20	20.01	Non-inferiority not demonstrated
	Chlorhexidine spray	23.61 ± 9.21						
2 (day 10)	Clove essential oil spray	59.03 ± 4.01	−12.92	5.56	2.33	−0.16	11.27	Non-inferior
	Chlorhexidine spray	64.58 ± 2.41						
3 (day 15)	Clove essential oil spray	100.00 ± 0.00	−20.00	0.00	0.00	NA	NA	Non-inferior
	Chlorhexidine spray	100.00 ± 0.00						

CIS = Clinical index score; CI = Confidence interval; SE = Standard error; NA = Not applicable. NA indicates that the statistic could not be calculated because both groups had zero variance.

A similar pattern was observed in the *M. canis* dermatophytosis model. The non-inferiority criterion was not met at day 5 but was achieved by day 10, when the lower bound of the 95% CI exceeded the prespecified margin. Non-inferiority was maintained at the final follow-up on day 15 (Table 5).

**Table 5:** Non-inferiority analysis of clove essential oil spray versus ketoconazole spray in the Microsporum canis dermatophytosis model.

**Follow-up**	**Treatment**	**%CIS reduction (mean ± SD)**	**Non-inferiority margin**	**Mean difference**	**SE of difference**	**95% CI, lower**	**95% CI, upper**	**Result**
1 (day 5)	Clove essential oil spray	27.78 ± 6.41	−6.67	5.56	5.56	−8.04	19.15	Non-inferiority not demonstrated
	Ketoconazole spray	33.33 ± 9.07						
2 (day 10)	Clove essential oil spray	55.56 ± 9.07	−12.22	5.56	5.56	−8.04	19.15	Non-inferior
	Ketoconazole spray	61.11 ± 6.42						
3 (day 15)	Clove essential oil spray	80.56 ± 5.56	−16.67	2.78	4.24	−7.60	13.16	Non-inferior
	Ketoconazole spray	83.33 ± 6.42						

CIS = Clinical index score; CI = Confidence interval; SE = Standard error.

In the *M. pachydermatis* *Malassezia* dermatitis model, the clove essential oil spray met the non-inferiority criterion relative to ketoconazole from the first follow-up on day 5 and maintained non-inferiority at days 10 and 15 (Table 6). Thus, by the second follow-up on day 10, the clove essential oil spray satisfied the prespecified non-inferiority criterion in all three experimental infection models.

**Table 6:** Non-inferiority analysis of clove essential oil spray versus ketoconazole spray in the Malassezia pachydermatis dermatitis model.

**Follow-up**	**Treatment**	**%CIS reduction (mean ± SD)**	**Non-inferiority margin**	**Mean difference**	**SE of difference**	**95% CI, lower**	**95% CI, upper**	**Result**
1 (day 5)	Clove essential oil spray	26.04 ± 9.84	−7.57	11.81	6.73	−4.65	28.26	Non-inferior
	Ketoconazole spray	37.85 ± 9.17						
2 (day 10)	Clove essential oil spray	61.46 ± 7.89	−12.36	0.35	4.57	−10.83	11.52	Non-inferior
	Ketoconazole spray	61.81 ± 4.61						
3 (day 15)	Clove essential oil spray	97.72 ± 5.56	−20.00	2.78	2.78	−4.02	9.57	Non-inferior
	Ketoconazole spray	100.00 ± 0.00						

CIS = Clinical index score; CI = Confidence interval; SE = Standard error.

### Dermal irritation assessment

Dermal tolerability of the clove essential oil spray was evaluated by monitoring erythema and edema at treated skin sites. No observable signs of dermal irritation were detected during the assessment period. Erythema and edema scores remained 0/4 at all evaluation points, including the final assessment performed 24 h after the last application on day 4 (Figure 6). Under the conditions of this experiment, repeated topical application of the 2.5% w/w clove essential oil spray therefore produced no detectable erythema or edema.

**Figure 6:**
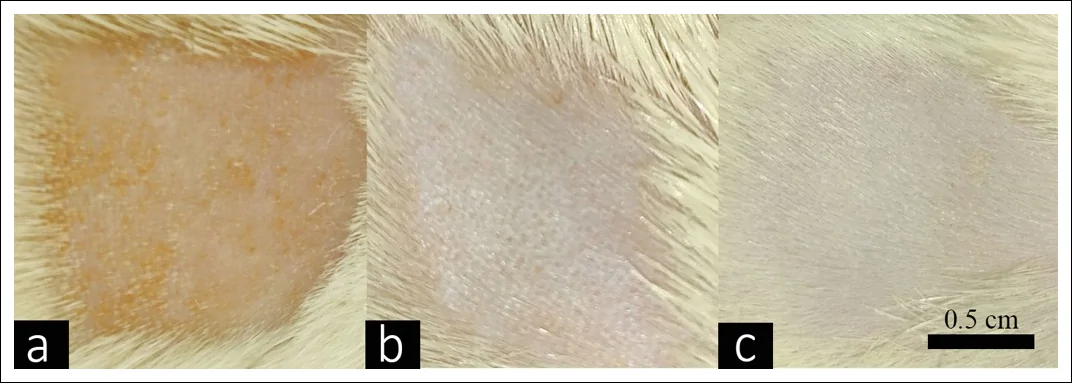
Dermal irritation assessment of clove essential oil spray in rats. (a) Baseline skin appearance before treatment; (b) skin appearance 24 h after the final application of normal saline (negative control) on day 4; and (c) skin appearance 24 h after the final application of clove essential oil spray on day 4. No erythema or edema was observed in either the negative control or clove essential oil spray group.

## DISCUSSION

### Therapeutic efficacy and non-inferiority of clove essential oil spray

The present study provides a pilot multi-pathogen non-inferiority evaluation of a topical clove essential oil spray in rat models of infectious dermatitis and extends previous *in vitro* observations to an *in vivo* experimental setting. The formulation demonstrated therapeutic activity across pyoderma caused by *S. pseudintermedius*, *Malassezia* dermatitis caused by *M. pachydermatis*, and dermatophytosis caused by *M. canis*. By day 10, the clove essential oil spray met the predefined non-inferiority criterion relative to the corresponding standard treatments in all three models. In the *S. pseudintermedius* and *M. pachydermatis* models, treatment was also associated with complete pathogen reduction by day 10, whereas progressive clinical improvement was observed across all three models.

The temporal pattern of non-inferiority differed among the infection models. In the *S. pseudintermedius* pyoderma and *M. canis* dermatophytosis models, the clove essential oil spray did not meet the prespecified non-inferiority criterion at the first follow-up on day 5 but achieved non-inferiority by day 10 and maintained it through day 15. In contrast, non-inferiority to ketoconazole in the *M. pachydermatis* model was achieved from day 5 and maintained throughout the treatment period. These findings indicate that the onset of measurable therapeutic response differed among the pathogen-specific models. However, the present study was not designed to determine the biological or pharmacokinetic mechanisms responsible for these temporal differences, which require further investigation.

The formulation characteristics may have contributed to topical delivery of the active constituents. Isopropyl myristate was incorporated as a carrier and Tween-80 as a surfactant. Isopropyl myristate has been reported to modify stratum corneum lipid organization and enhance penetration of compounds through the skin [31]. Nevertheless, the present study did not directly assess dermal penetration, tissue concentrations, or pharmacokinetic behavior of eugenol or other constituents. Therefore, the current findings cannot establish the contribution of individual excipients to the observed therapeutic response.

Previous studies have evaluated clove essential oil or eugenol-based preparations against dermatological infections in cattle, buffaloes, and guinea pigs [13–15]. The present study extends this evidence by applying a standardized non-inferiority framework and evaluating clinical and microbiological outcomes across bacterial, yeast, and fungal experimental infection models. This approach enabled direct comparison of the developed formulation with chlorhexidine for bacterial infection and ketoconazole for yeast and fungal infections under controlled experimental conditions.

### Potential antimicrobial mechanisms of clove essential oil

The therapeutic activity observed in the present study is consistent with the established antimicrobial properties of clove essential oil. Previous characterization of clove essential oil from the same source identified eugenol as the predominant constituent (98.87%), with trans-caryophyllene accounting for the remaining 1.13% [10]. The predominance of eugenol is relevant because this compound has demonstrated antimicrobial activity against a broad range of microorganisms.

Several mechanisms have been proposed to explain the antimicrobial activity of clove essential oil and eugenol. These include disruption of the microbial cell wall and cytoplasmic membrane, increased membrane permeability, and subsequent leakage of intracellular components, including proteins and nucleic acids. Eugenol has also been reported to promote oxidative stress by generating reactive oxygen species, potentially overwhelming microbial antioxidant defense mechanisms. At the molecular level, eugenol may interact with enzymes involved in essential metabolic pathways, including enoyl-[acyl-carrier-protein] reductase, thereby interfering with fatty acid biosynthesis and cellular energy metabolism [9]. These multiple antimicrobial mechanisms may explain the broad activity of clove essential oil against the different microorganisms evaluated in this study.

Trans-caryophyllene was present at a considerably lower concentration than eugenol. Although it may contribute to the biological activity of clove essential oil, the present study did not independently evaluate the antimicrobial activity of trans-caryophyllene or interactions between individual constituents. Therefore, the current experimental data cannot determine possible additive or synergistic effects among eugenol, trans-caryophyllene, and other formulation components.

### Additional pharmacological properties and dermal tolerability

Beyond its antimicrobial activity, clove essential oil has been reported to possess antioxidant, anti-inflammatory, immunomodulatory, and wound-healing properties that may be relevant to dermatological applications. Previous studies have demonstrated antioxidant and wound-healing activities of clove oil formulations [32], and other investigations have described reductions in oxidative stress associated with clove essential oil-based preparations [33]. Anti-inflammatory and immunomodulatory effects of clove essential oil and eugenol have also been associated with modulation of neutrophil function and inflammatory responses [34]. In addition, previous studies of clove oil-based topical formulations have reported beneficial effects in experimental inflammatory skin conditions [35].

These pharmacological properties provide plausible biological context for the progressive improvement in lesion appearance observed during treatment. However, the present study did not measure inflammatory biomarkers, oxidative stress indices, wound-healing parameters, nociception, or immune responses. Consequently, the current data cannot distinguish the relative contribution of antimicrobial, anti-inflammatory, antioxidant, or wound-healing effects to clinical improvement.

Dermal tolerability is particularly important for topical treatments because local irritation may exacerbate existing skin lesions and compromise treatment adherence. In the present study, repeated topical application of the 2.5% w/w clove essential oil spray produced no observable erythema or edema, with both parameters remaining at 0/4 throughout the dermal irritation assessment. These findings indicate favorable local tolerability under the specific experimental conditions and application period evaluated. Nevertheless, absence of observable irritation during short-term exposure does not establish long-term dermal safety, particularly in naturally occurring chronic dermatological disease.

### Strengths, limitations, and future perspectives

A major strength of this pilot study was the use of three pathogen-specific experimental models encompassing bacterial, yeast, and fungal causes of infectious dermatitis. Including corresponding standard treatment groups enabled direct non-inferiority comparisons with chlorhexidine and ketoconazole, while simultaneous assessment of clinical response and microbiological outcomes provided complementary measures of therapeutic efficacy. Blinded outcome assessment and standardized CIS criteria further strengthened the evaluation.

Several limitations should, however, be considered when interpreting the findings. First, this was a pilot study with a small sample size of four animals in each active-treatment group and two animals in each negative control group within each pathogen model. Although the sample size was based on prespecified non-inferiority assumptions, the limited number of animals restricts the precision and generalizability of the findings. Second, the infections were experimentally induced in rats and represented relatively acute disease models. These conditions may not fully reproduce the complexity, chronicity, host factors, concurrent disease, or environmental influences associated with naturally occurring pyoderma, *Malassezia* dermatitis, and dermatophytosis in dogs and cats. Differences in skin structure and permeability between rats and the intended target species should also be considered when extrapolating these results.

Third, the study evaluated only one concentration of clove essential oil (2.5% w/w) over a relatively short 15-day treatment period. Therefore, the optimal concentration, dose–response relationship, minimum effective concentration, and effects of prolonged treatment remain unknown. The study did not perform detailed histopathological evaluation, including epidermal thickness, inflammatory cell infiltration, and tissue repair. The study also did not evaluate systemic toxicity, circulating biomarkers, dermal absorption, pharmacokinetics, or the independent effects of the formulation vehicle. Furthermore, although microbial clearance was assessed, the study did not investigate whether repeated exposure to the formulation affected antimicrobial susceptibility or the emergence of resistance.

Future studies should therefore evaluate larger experimental populations and multiple formulation concentrations to establish dose–response relationships and optimize treatment regimens. Histopathological assessment, inflammatory and oxidative stress biomarkers, dermal penetration studies, systemic safety evaluation, and longer follow-up periods would provide a more comprehensive assessment of therapeutic mechanisms and safety. Formulation stability under defined storage conditions should also be examined before broader clinical application. Most importantly, randomized controlled clinical trials in dogs and cats with naturally occurring pyoderma, *Malassezia* dermatitis, and dermatophytosis are required to determine whether the efficacy and dermal tolerability demonstrated in these experimental rat models can be reproduced in the intended veterinary patient populations.

## CONCLUSION

This pilot study demonstrated that a topical 2.5% w/w clove essential oil spray was therapeutically effective and well tolerated in experimental rat models of pyoderma caused by *S. pseudintermedius*, *Malassezia* dermatitis caused by *M. pachydermatis*, and dermatophytosis caused by *M. canis*. By day 10, the formulation met the prespecified non-inferiority criterion relative to chlorhexidine or ketoconazole in all three infection models. At day 15, CIS reduction reached 100% for *S. pseudintermedius*, 97.72% for *M. pachydermatis*, and 80.56% for *M. canis*. Complete pathogen reduction was achieved for *S. pseudintermedius* and *M. pachydermatis*, whereas 75% culture negativity was achieved for *M. canis*. Repeated topical application also produced no observable erythema or edema, supporting favorable short-term dermal tolerability under the tested experimental conditions.

From a practical perspective, the ready-to-use spray formulation offers a potentially convenient approach for localized topical administration and warrants further development as an alternative topical strategy for veterinary infectious dermatitis. Its activity across bacterial, yeast, and fungal experimental infections is particularly relevant to developing topical formulations that can address different etiological agents. However, these findings represent proof-of-concept evidence from a small experimental rat study and should not yet be extrapolated directly to clinical efficacy in dogs, cats, or other target animals. Larger studies should evaluate dose–response relationships, prolonged treatment and follow-up, histopathological changes, dermal penetration, systemic safety, formulation stability, vehicle effects, and the potential for changes in microbial susceptibility. Randomized controlled clinical trials in naturally infected companion animals are ultimately required to confirm therapeutic efficacy, optimal treatment regimens, and long-term safety. Overall, the findings provide a foundation for the further development and clinical evaluation of clove essential oil spray as a topical therapeutic candidate for infectious dermatitis in veterinary medicine.

## DATA AVAILABILITY

The data supporting the findings of this study are available from the corresponding author upon reasonable request.

## GENERATIVE AI DECLARATION

The authors declare that generative artificial intelligence (AI) tools were used solely to improve language, grammar, and readability during manuscript preparation. All scientific content, data analysis, interpretation of results, and conclusions were entirely developed and verified by the authors. The authors take full responsibility for the accuracy, integrity, and originality of the work presented.

## AUTHORS’ CONTRIBUTIONS

ET: Designed the experiments, performed pathogen confirmation and quantification and clinical evaluation, assisted with the induction of experimental dermatitis, analyzed the data, and drafted and edited the manuscript. GNB: Contributed to formulation preparation and experimental design and reviewed and edited the manuscript. RS: Assisted with microbial inoculum preparation, culture, and quantification and contributed to data analysis. KS: Managed animal care, performed the induction of experimental dermatitis and dermal irritation assessment, conducted clinical evaluations, and collected the data. JA: Conceived, designed, and supervised the study, developed and prepared the clove essential oil spray formulation, performed microbial inoculum preparation and treatment procedures, validated the data, and reviewed and edited the manuscript. All authors have read and approved the final manuscript.
